# Flow cytometry optimizing the diagnostic approach in inborn errors of immunity: experience from Egypt

**DOI:** 10.1186/s13223-022-00688-w

**Published:** 2022-06-02

**Authors:** Safa Meshaal, Rabab EI Hawary, Alia Eldash, Aya Erfan, Dalia Abd Elaziz, Radwa Alkady, Sohilla Lotfy, Nermeen Galal, Jeannette Boutros, Aisha Elmarsafy

**Affiliations:** 1 Clinical and Chemical Pathology Department, Faculty of Medicine, Cairo University, Cairo, 11562 Egypt; 2 Pediatric Department, Faculty of Medicine, Cairo University, Cairo, Egypt

**Keywords:** Flow cytometry, Inborn errors of immunity, Intracellular proteins, Primary immunodeficiency

## Abstract

**Background:**

Human inborn errors of immunity (IEI) are a group of inherited genetic disorders of the immune system. IEI Patients suffer from severe repeated infections, autoimmunity, lymphadenopathy and/or increased susceptibility to malignancies. IEI are due to absence, disproportion, or loss of function of immune cells; mostly inherited in autosomal recessive manner, hence are more common in countries with high rate of consanguinity. Definite diagnosis of IEI is achieved by genetic analysis, however it is not always available. Aim: to report on different IEI categories and impact of expanding the use of flow cytometry (FCM) in diagnosis, categorization and follow up of IEI patients in a highly consanguineous population.

**Methods:**

Retrospective chart review on different IEI categories diagnosed at the primary immunodeficiency center in Cairo University Specialized Pediatric hospital from 2011 to 2021 based on expanding the use of FCM.

**Results:**

1510 IEI patients were diagnosed; 480 were diagnosed genetically with FMF, 11 with cystic fibrosis and 1019 patients were diagnosed with other IEI disorders. Phagocytic defects were the commonest (30%) followed by severe combined immunodeficiency (22%) and combined immunodeficiency (18.3%). FCM testing properly diagnosed and categorized 73% of the cases.

**Conclusion:**

Using multi-color FCM to evaluate immune cells populations, subpopulations, functions, and intracellular proteins expression is proved a useful cost-effective method for screening, categorization and follow up of IEI patients. FCM can improve the diagnosis of IEI significantly when tests are properly targeted and well designed. This study presents a 10-year experience in diagnosis of IEI using FCM at a tertiary referral center in a setting of limited resources and yet high prevalence of IEI.

**Supplementary Information:**

The online version contains supplementary material available at 10.1186/s13223-022-00688-w.

## Introduction

Human inborn errors of immunity (IEI)/Primary immunodeficiencies (PIDs) are a group of heterogeneous disorders with underlying monogenic defects affecting the immune system [1]. Patients suffer from repeated life-threatening infections that necessitate early diagnosis and management [2, 3]. IEI were used to be considered as rare diseases which affect around 1:10,000 births [4]. However, with the continuous discovery of new genes involved in IEI and better understanding of different phenotypes, the collective prevalence of IEI is expected to be around 1:1000/1:5000 [1]. The latest report by the International Union of Immunological Societies (IUIS) Expert Committee, described 430 different disorders of IEI subdivided into ten categories, with a brief update published in 2021 adding 26 more genes to the list [5, 6]. Most of these disorders have an autosomal recessive (AR) mode of inheritance which makes them more common in consanguineous populations from Middle East and North Africa (MENA) [7, 8]. Although, definitive and prenatal diagnosis are achieved by molecular analysis, however, many countries with high prevalence of IEI including Egypt have limited accessibility to genetic testing [3, 7, 9, 10]. Another important challenge in the diagnosis of IEI, is the phenotypic heterogeneity despite the same genetic defect. This heterogeneity makes targeted genetic diagnosis difficult and urges extensive, rapid, and yet easy-to perform tests for proper evaluation and categorization of patients [11–13].

Flow cytometry (FCM) has gained great importance in the diagnosis and follow up of different PID disorders (PIDDs) [11, 14–16]. As most of PIDDs are due to either absence, disproportions or dysfunction of immune cells or the proteins expressed by these cells, such peculiar features can be analyzed extensively by multi-color FCM [15]. Several studies have described the role of FCM in the diagnosis of PIDDs, however few presented particular centers' experiences, and none was from MENA [11, 14, 15, 17, 18]. The PID center at Cairo University (CU) Specialized Pediatric Hospital is the largest tertiary referral center in Egypt receiving PID patients from all over Egypt and nearby Arab countries. In this study, we present the 10-years' experience of the only specialized PID lab in Egypt, highlighting the impact of expanding the use of FCM on diagnosis, categorization and follow up of IEI patients.

## Methods

The study was conducted at the PID center in CU Specialized Pediatric hospital and was approved by the Institutional Review Board of Kasr Al-Ainy faculty of Medicine-Cairo University. The study was designed as a retrospective chart review through 10 years from 2011 to 2021 and included patients who were diagnosed with a specific IEI category according to the European society for immunodeficiency (ESID) diagnostic criteria and IUIS classification [5, 19]. Patients diagnosed with Familial Mediterranean Fever and cystic fibrosis were excluded.

Patients were screened for PID disorders when having manifestations suggestive of immune deficiency including repeated, unusual, or severe infections, autoimmunity, lymphadenopathy and/or immune dysregulation like cytopenia, early-onset inflammatory bowel disease and/or endocrinopathy. Screening and categorization of patients was done based on the clinical picture and flow cytometric analysis.

Peripheral blood samples were received from patients evaluated by the PID clinic in the hospital and patients admitted in the hospital's different wards and ICUs. The center received samples from all over Egypt which were analyzed on the same day of collection, as usually the patients come to the clinic for clinical evaluation and laboratory testing. Special arrangements were done for patients admitted in hospitals in other governorates where usually physicians communicate with the lab to deliver the required samples within 24 h of collection.

Immunophenotyping of peripheral blood lymphocytes' subsets including CD3 as pan-T marker, CD4 for T-helper, CD8 for T-cytotoxic, CD19 for B cells and CD56/CD16 for NK cells is the initial screening investigation. lymphocytes were characterized by forward and side scatter and expression of CD45.

**Fig. 1:**
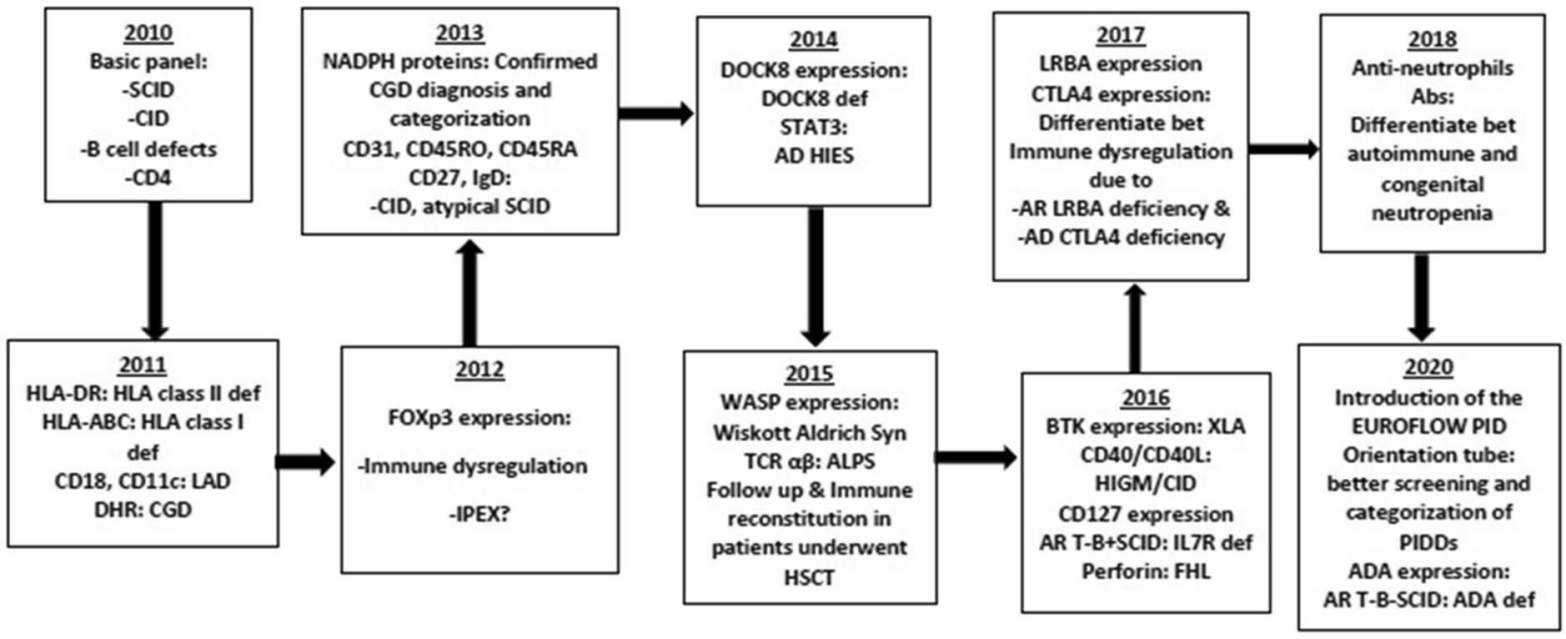
Timeline showing establishment and introducing new flow cytometry tests along the years

Between 2010 and 2021, many tests have been introduced and optimized for different PIDDs diagnosis. Extended panels, functional assays and intracellular staining of different proteins were added to the diagnostic and screening tests along the years (Fig. 1).

In 2020, the eight-colors FACS Canto-II was installed in our laboratory and thus we started to apply the EuroFlow PID orientation [20].

Most of the monoclonal antibodies, were titrated to estimate the proper concentration to be used and usually, the used concentration is less than advised by the manufacturer. We used some purified monoclonal antibodies that were not previously tested on FCM. In these experiments, careful titration was done, and test results were confirmed by targeted genetic analysis, until optimization of the FCM test was achieved [12, 21, 22].

## Diagnosis of immunodeficiencies affecting cellular and humoral immunity

### Severe combined immunodeficiency (SCID)

**Fig. 2:**
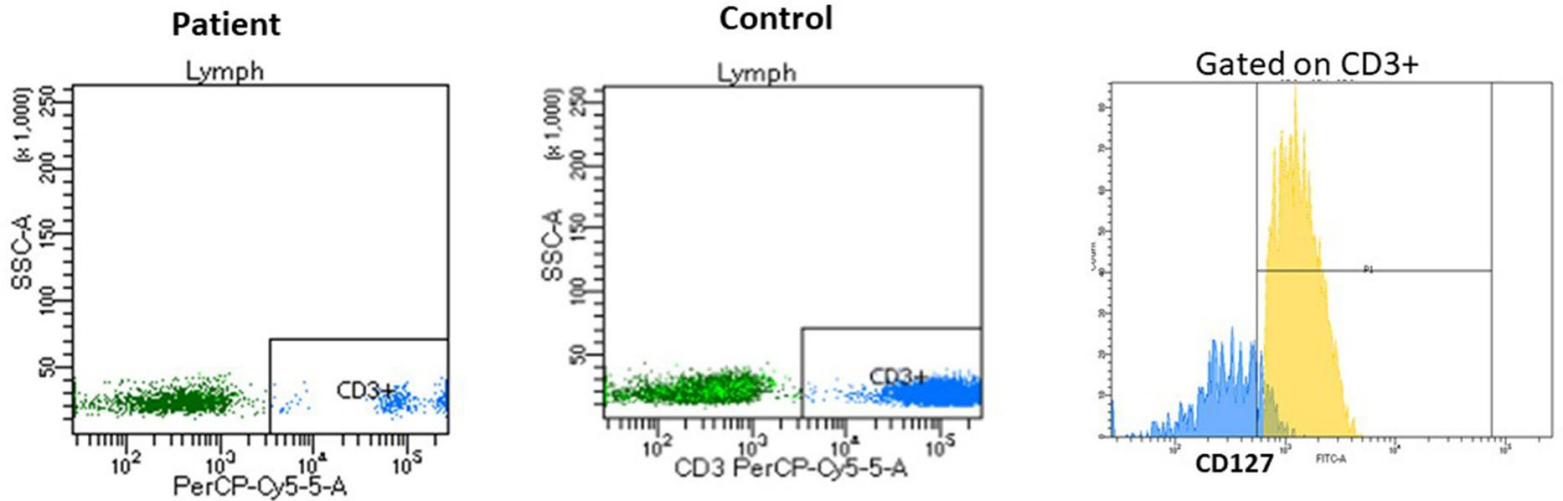
Overlay of CD127 expression gating on CD3+ cells in a T-B+SCID patient (in blue) and healthy control (in yellow) showing defective CD127 expression on the residual CD3+ cells in the patient

**Fig. 3:**
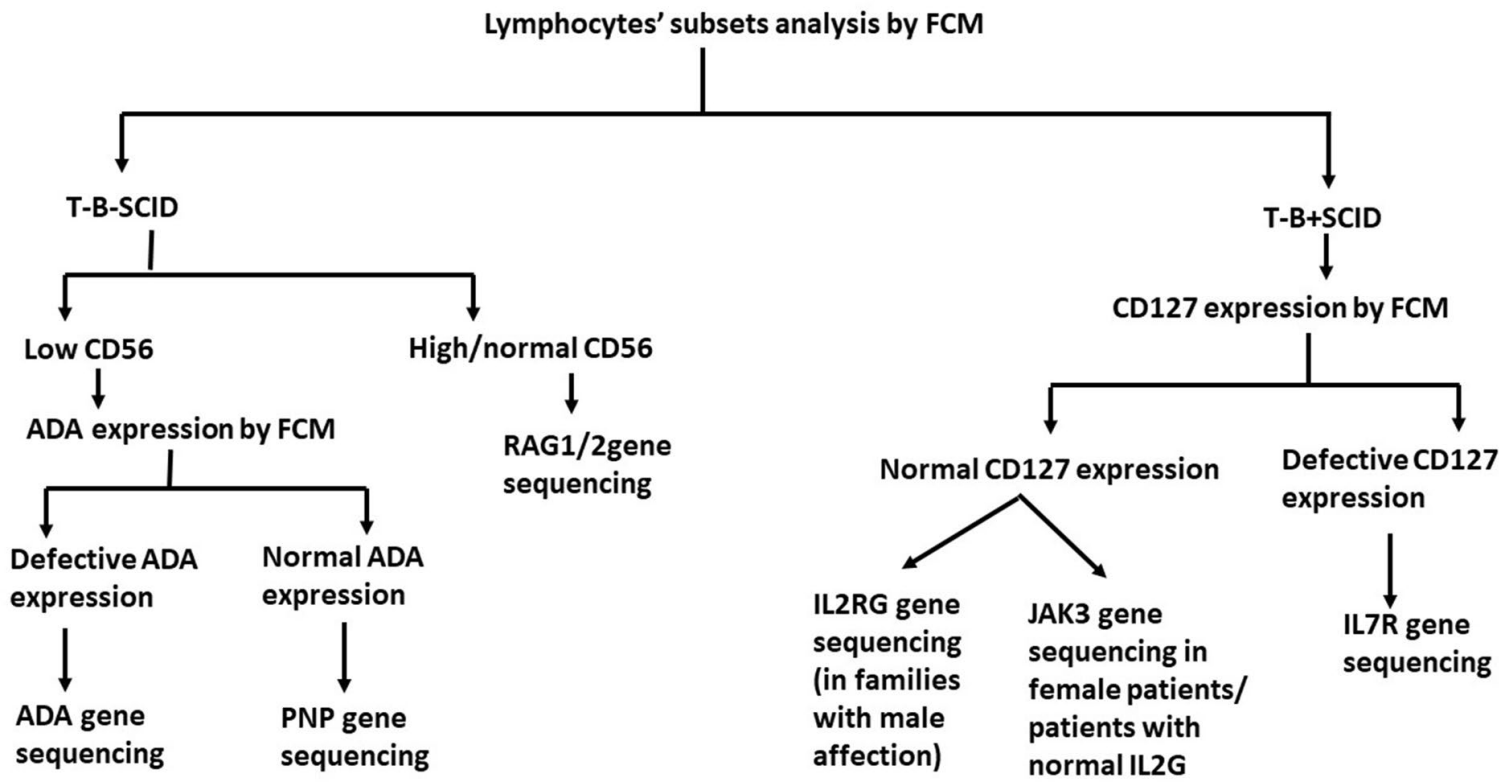
Algorithm illustrating the approach of using flow cytometry assays to diagnose and categorize T-B-SCID and T-B+SCID patients based on preservation of NK, CD127 and ADA expression

According to the presence or absence of the B cells and NK cells; SCID patients were divided into T-B-NK+SCID, T-B-NK-SCID, T-B+NK+SCID, T-B+NK-SCID [23]. For T-B+SCID cases, IL-7RA (CD127) expression was evaluated by FCM gating on the small population of CD3+T cells (Fig. 2). Patients with defective CD127 expression, are then tested by targeted *IL7R* gene sanger sequencing, while T-B+SCID patients with normal CD127 expression are tested by Sanger sequencing for *JAK3* and/or *IL2RG* gene based on gender of the patient, family history, and consanguinity. T-B-NK+SCID are tested for *RAG1/2* variants by Sanger sequencing and those with negative results can be tested by next generation sequencing (NGS). T-B-NK-SCID cases are tested for ADA expression by FCM gating on the total lymphocytes [24]. Those with ADA deficiency are genetically tested for *ADA* gene variants while those with normal ADA expression are tested for *PNP* variants (Fig. 3).

### Combined immunodeficiency (CID) less profound than SCID

#### Dedicator of cytokinesis 8 (*DOCK8) deficiency*

**Fig. 4:**
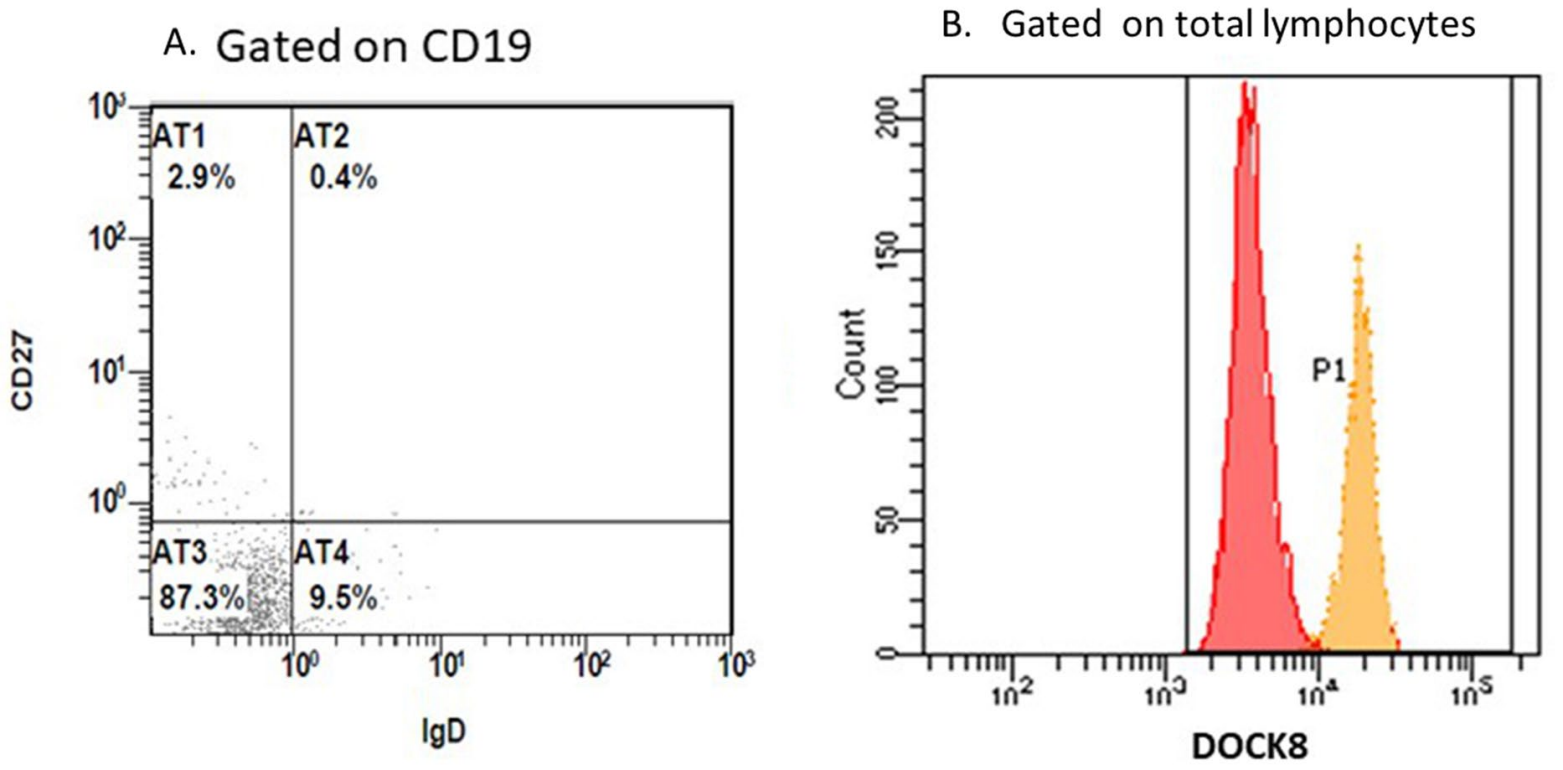
Histograms showing the Flow cytometry assay of a DOCK8 deficiency patient: A. gating on B cells: 87.3% of the B cells were CD27-IgD−, B. An overlay of the DOCK8 expression of the patient (in red) and the control (in orange)

In 2014, DOCK8 expression by FCM was started in our lab. Definitive diagnosis of DOCK8 deficiency can be achieved when the patient has 4 laboratory criteria in the presence of suggestive clinical presentation (Fig. 4) [21]:

1. Defective DOCK8 expression by FCM compared to the control sample analyzed on the same day.
2. CD4 lymphopenia.
3. Arrest of B cell maturation (mostly CD19+CD27−IgD−).
4. Profound defect in Th17 cells following invitro stimulation.

### Major histocompatibility complex class II (MHC II) deficiency

In patients with CD4 lymphopenia and CID feature, HLA-DR expression is assessed on B cells as they are the most abundant antigen presenting cells (APCs) in peripheral blood.

### CD40/CD40L deficiency

Patients with elevated serum IgM are investigated for CD40 L deficiency and/or CD40 deficiency according to the gender and family history. CD40 L (CD154) expression is evaluated on activated CD4+ cells after MNCs are stimulated by phorbol myristate acetate (PMA)/Ionomycin for 4 h. CD40 expression is assessed on CD19+B cells without prior need for stimulation as it is constitutively expressed on the B cell surface.

### Isolated CD4 lymphopenia

CID Patients having CD4 lymphopenia are tested for HLA-DR, DOCK8 expression and T cell maturation and recent thymic immigrants (CD4+CD45RA+CD31+). CD45RA was used as a marker for naïve T cells while CD45RO was used as a marker for memory T cells.

## Diagnosis of CID associated with syndromic features

### Wiskott aldrich syndrome (WAS)

WASp expression by FCM is a simple intracellular staining test performed on whole blood. It is evaluated on lymphocytes as percentage and MFI and compared to the control done on the same day and the background staining of each sample.

We used purified mouse anti-human WASP monoclonal antibodies and secondary FITC-conjugated goat anti-mouse IgG/IgM to perform the test.

### STAT3 deficiency

For patients suspected to have STAT3 deficiency, phosphorylation of STAT3 was evaluated after stimulation of MNC by IL-6 followed by fixation and permeabilization of the cells and incubation with anti-PSTAT3. Th17 cells are also evaluated following stimulation of MNC with stimulation cocktail (PMA/Ionomycin/brefeldin). and expressed as percent of cells expressing IL-17 within memory CD4+CD45RO+T cells compared to the control.

## Diagnosis of immunodeficiencies with predominantly antibodies defect

### X-linked agammaglobulinemia (XLA)

BTK expression analysis was done for the patients and their mothers by FCM by gating on the monocytes. Monocytes were characterized by forward and side scatter and expression of CD14. XLA patients showed absent or very low (dim) BTK expression, while their mothers showed bimodal pattern of the carrier on monocytes and normal expression on B cells. For each test, a control sample is run for comparison. The percentages and MFI of BTK on B and CD14+ monocytes are assessed.

### Common variable immunodeficiency (CVID)

For patients with CVID picture, B cell maturation is assessed; naïve (CD27−IgD+), non-switched memory (IgD+CD27+), class-switched memory (CD27+IgD−), as well as CD4+and CD8+T cell differentiation. LRBA and CTLA4 expression are done for suspected cases.

## Diagnosis of diseases of immune dysregulation

### Lipopolysaccharide (LPS)-responsive and beige-like anchor protein (LRBA) deficiency

**Fig. 5:**
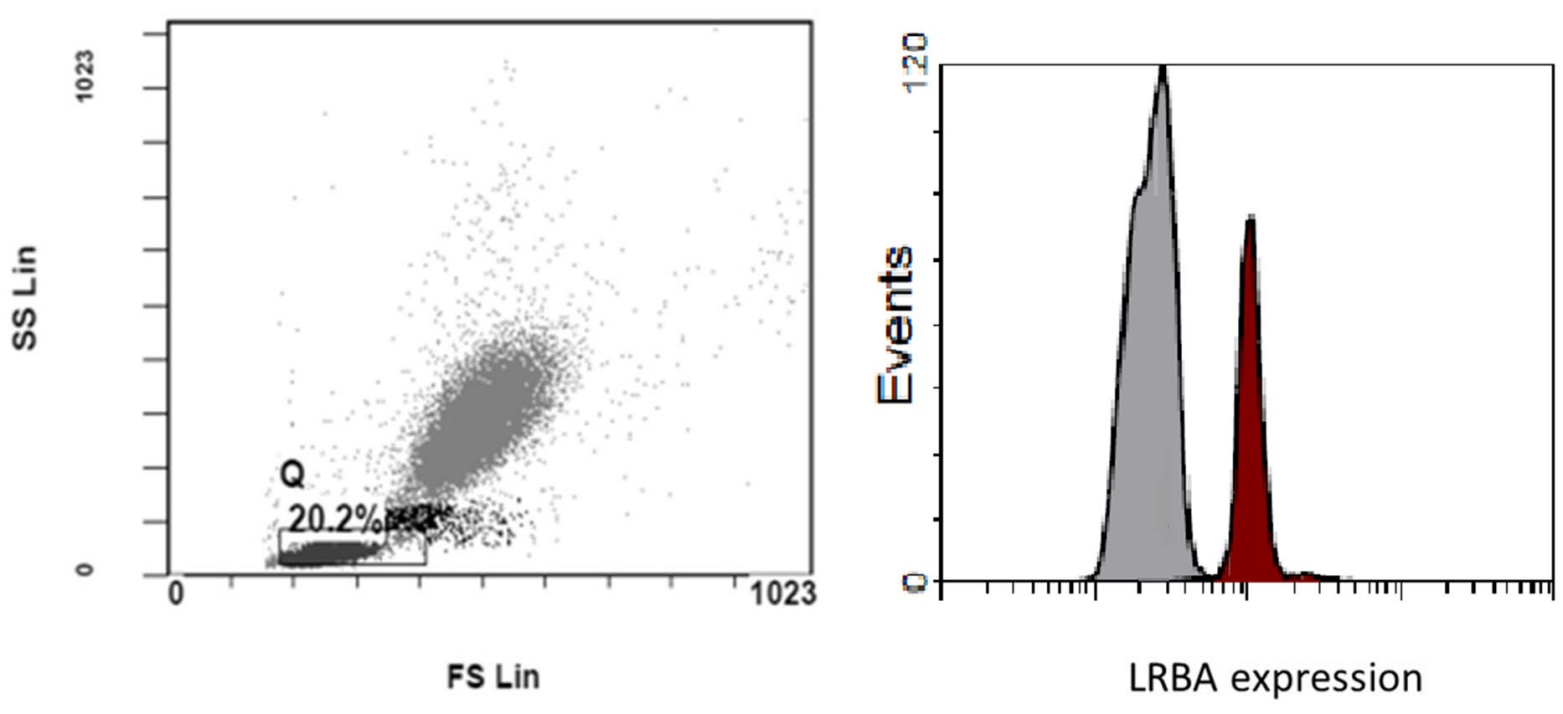
Histogram showing LRBA expression gating on the total lymphocytes with overlay of the control (in brown) over the patient (in grey)

Percentage and MFI of intracellular LRBA protein expression is compared to the control done with each tested sample. MFI index was calculated by dividing MFI of the stained sample by that of the background staining tube. Other immunological biomarkers that would help confirm the diagnosis included low B cells percentages and an increase in memory CD4+CD45RO+T cells (Fig. 5) [12].

### Cytotoxic t lymphocytes antigen 4 (CTLA4) deficiency

**Fig. 6:**
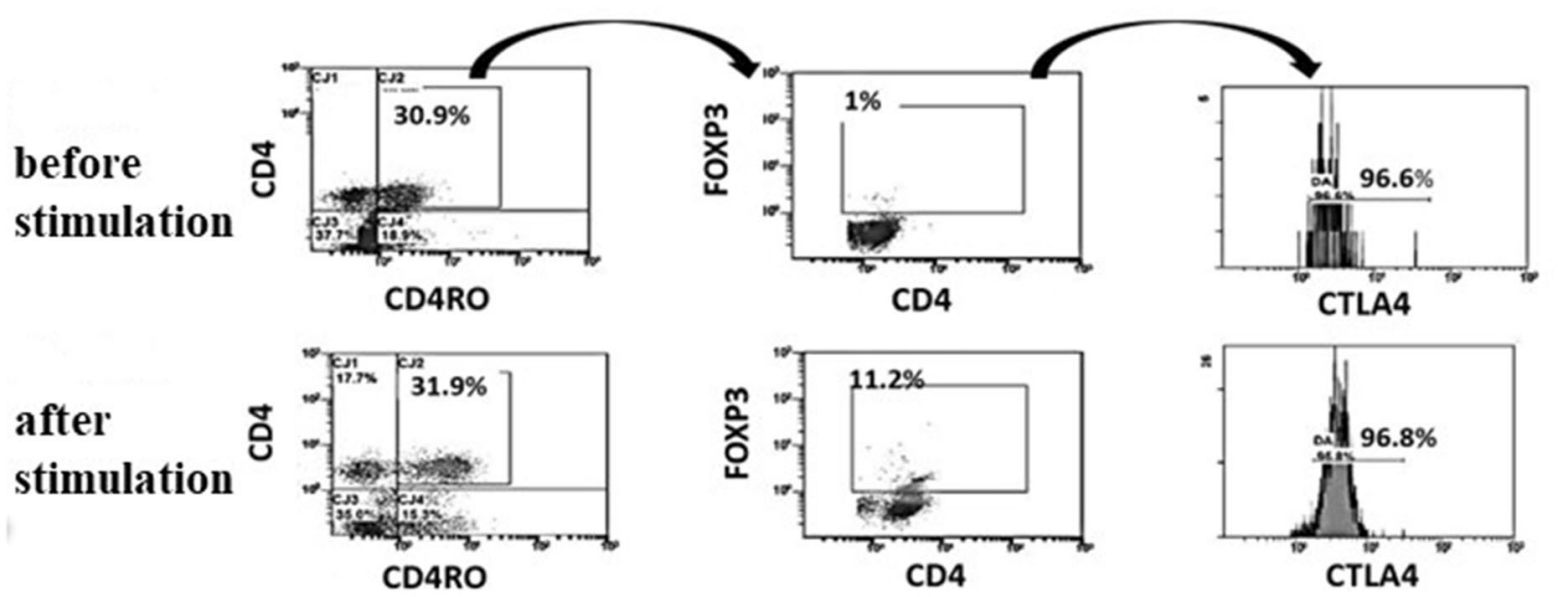
Histogram showing the gating strategy to assess the CTLA4 expression in a healthy control before and after stimulation of T cells. The CTLA4 is assessed within the memory T regs (CD4+CD45RO+FoxP3+cells)

As resting T cells express low level of CTLA4, proper assessment of CTLA4 expression, requires stimulation of MNCs with PMA/Ionomycin. CTLA4 percentage and MFI were evaluated in CD4 cells and Foxp3+Tregs and compared to the control. We also compared the level of expression of CTLA4 before and after the stimulation [12]. T lymphocytes from patients with CTLA4 deficiency fail to express CTLA4 protein in response to invitro stimulation when gating on memory CD45RO+Foxp3+Tregs (Fig. 6).

### Autoimmune lymphoproliferative syndrome (ALPS)

Gating on CD3+T cells, expression of TCRαβ, CD4 and CD8 were evaluated. Pathological expansion of double negative T cells is defined as CD4-CD8-TCRαβ^**+**^ > 2.5% of T cells [14].

### Perforin deficiency

In our lab, perforin expression was assessed in CD56+NK cells and CD8‏+T cells in patients with picture suggestive of familial hemophagocytic lymphohistiocytosis.

## Diagnosis of defects of phagocytes

### Chronic granulomatous disease (CGD)

FCM has been used for functional assessment of the oxidative respiratory burst of granulocytes using the Dihydrorhodamine test (DHR) [21, 25].. In 2014, to achieve confirmed diagnosis of CGD, we introduced the assessment of intracellular NADPH components (gp91*phox*, p22*phox*, p46*phox* and p67*phox*) by FCM. For most of the cases, gp91*phox* deficiency is associated with p22*phox* deficiency. DHR test was done for mothers of male patients with deficiency of gp91*phox* and p22*phox* to differentiate between X-linked and AR forms as mothers showed bimodal pattern of the carrier in X-CGD.

### Leucocyte adhesion deficiency (LAD)

FCM was used for diagnosis of LAD I by assessing expression of CD18 & CD11 on the neutrophils. According to the level of expression of CD18, LAD is subclassified into severe (<2% of CD18− expressing neutrophils) and moderate (2–30%) forms [26].

### Congenital neutropenia

For patients with neutropenia, autoimmune neutropenia was excluded by FCM. Patient's peripheral blood was incubated with FITC-labelled Rabbit anti-human IgG for an hour. The cells were then washed to remove excess un-bound antibodies and analyzed by FCM. Neutrophils were characterized by forward and side scatter properties. Also, the serum of the patient was incubated for 2 h with pooled neutrophils from 5 different control samples. The sample were then washed, and FITC-labelled Rabbit anti-human IgG was added and incubated for 1 hour and then washed to remove excess unbound antibodies. The samples were then analyzed by FCM. Patients who did not have anti-neutrophil antibodies were then sent for genetic diagnosis of congenital neutropenia.

## Follow up of patients following hematopoietic stem cell transplantation (HSCT)

**Fig. 7:**
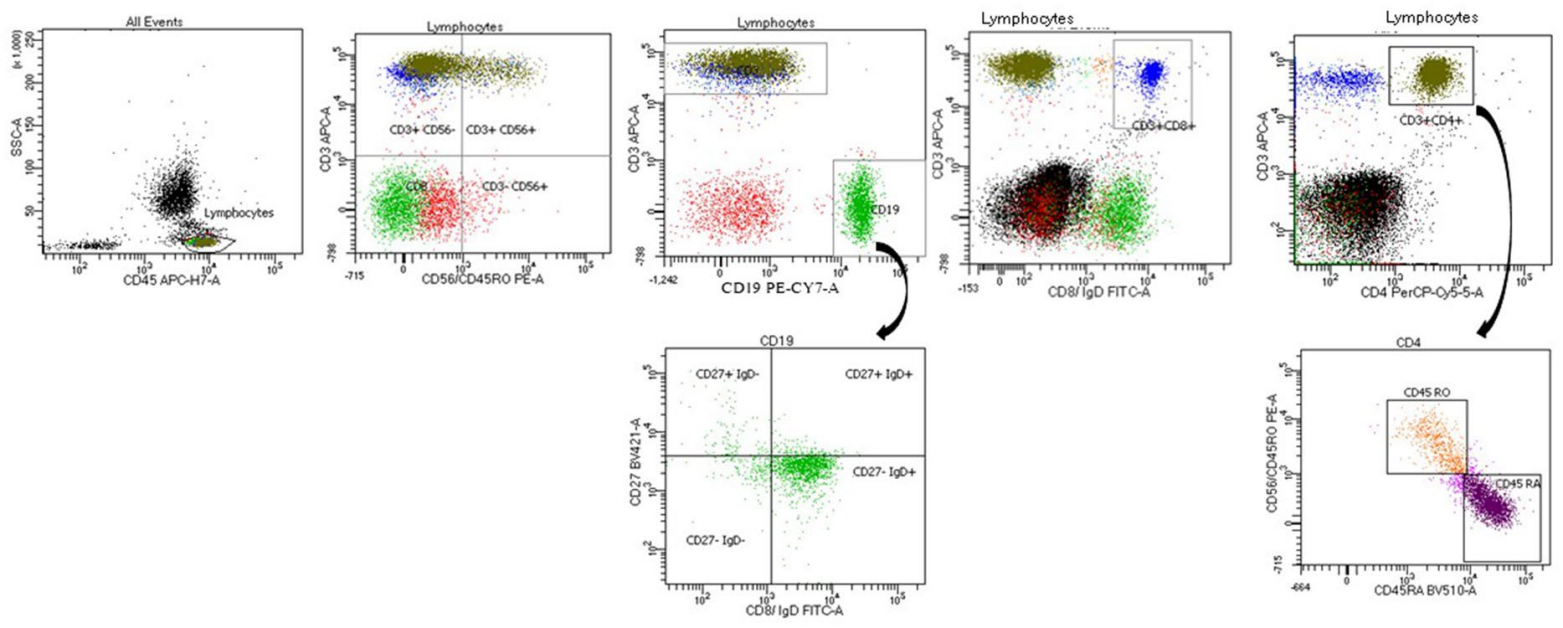
Histograms showing the gating strategy using 10 different monoclonals on the 8-color FACs CANTO-II for analysis of different lymphocytes subsets in a patient underwent HSCT. Lymphocytes are characterized by CD45 expression, forward and side scatter. APC-CD3 blotted against PE-CD56 for NK assessment. APC-CD3 blotted against PE-cy 7-CD19 for T and B cells identification. For T cytotoxic and T helper, APC-CD3 is blotted against FITC-CD8 and PerCP-Cy 5.5-CD4 respectively. Gating on CD4+, naïve and memory T helper are assessed by BV510-CD45RA and PE-CD45RO. Gating on CD19+, naïve and memory B cells are assessed by FITC-IgD and BV421-CD27

Laboratory testing, as part of the follow up for patients following HSCT, included assessment of different lymphocytes subsets, memory, naïve T & B lymphocytes, recent thymic emigrants, protein expression for the underlying defective protein e.g. DOCK8, LRBA, WASP (Fig. 7).

## Results

**Fig. 8:**
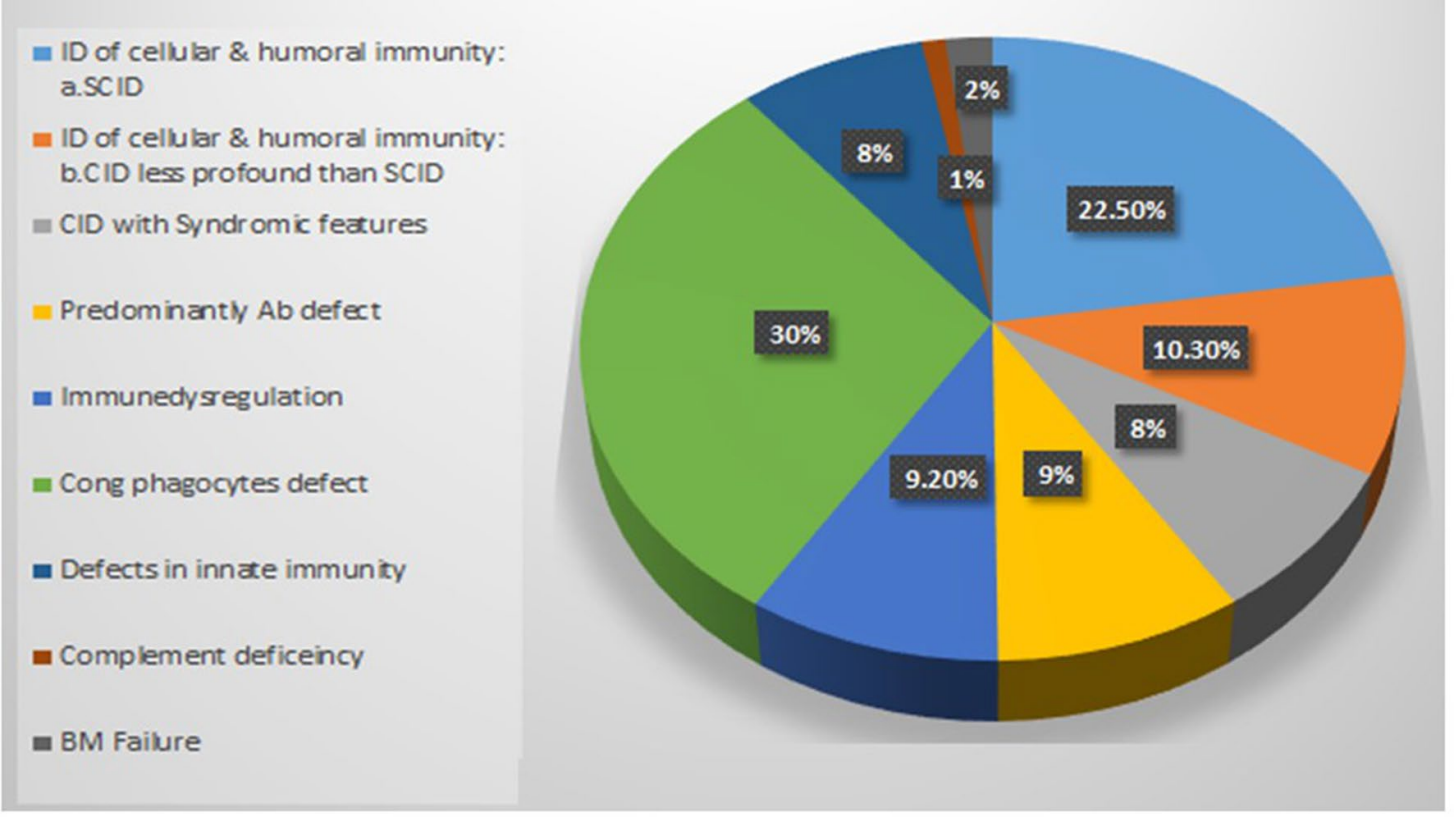
Percentages of IEI disorders by category in Egypt according to the IUIS classification 2019

During the period between 2011 and 2021, 1510 patients were diagnosed with IEI at our center. Four hundred-eighty had Familial Mediterranean Fever and 11 had cystic fibrosis and were excluded from the study as they have different service set ups. One thousand-nineteen patients (55% males and 45% females) were diagnosed with 50 different PID disorders in eight categories (Fig. 8). FCM helped to categorize and reach the diagnosis in 73% of the patients while 28% were categorized based on the clinical manifestations and genetic testing when available. Table 1 shows the increase in the number of the cases diagnosed in our center. Hereby we present the results of the patients screened and/or diagnosed based on FCM results.

**Table 1:** Main IEI categories/diseases which FCM improved their rate of diagnosis

Category	Disease	5 years	10 years
CID affecting cellular and humoral immunity: a SCID	T-B−SCID	46	140
b CID less profound than SCID	T-B+SCID	35	78
	DOCK8 deficiency	12	55
	Omenn syndrome	10	12
	MHC class II deficiency	3	22
	CD40L deficiency	1	3
CID with syndromic features	WAS	5	30
	STAT3	1	3
Immunodeficiencies with predominantly antibodies defect	XLA	14	22
Diseases of immune dysregulation:	LRBA	–	27
	CTLA4	–	1
	ALPS	4	11
	IPEX/IPEX-like	4	33
Congenital defects of phagocytes	CGD	45	205
	LAD	10	35
	Congenital neutropenia	3	34

### Severe combined immunodeficiency (SCID)/omenn syndrome

Two hundred-thirty SCID patients were diagnosed between 2011 and 2021. Ninety-six (42%) were females and 134 (58%) were males. One hundred-forty patients were diagnosed with T-B-SCID, 78 with T-B+SCID and twelve with Omenn Syndrome. Sanger sequencing was performed for patients based on FCM classification revealing 43 patients had RAG1/RAG2 deficiency, nine had ADA deficiency, 13 patients had JAK3 deficiency, eight had IL2RG deficiency and four had IL-7R deficiency.

### Combined immunodeficiency (CID) less profound than SCID

#### Dedicator of cytokinesis 8 (*DOCK8) deficiency*

Between 2014 and 2021; based on the FCM results, 55 patients were diagnosed with DOCK8 deficiency among 112 CID cases screened; 26 (47%) were females and 29 (53%) were males. Nevertheless, only one patient showed normal DOCK8 expression, yet had clinical manifestations and immunological biomarkers suggestive of DOCK8 deficiency. The patient's DNA was tested by WES which revealed an in-frame insertion which resulted in duplication of the amino acid Lysine at position 1955; a mutation which did not affect the integrity of the protein.

### Major histocompatibility complex class II (MHC II) deficiency

Twenty-two patients with MHC-II deficiency (16 males and 6 females) were diagnosed using FCM. Those patients presented with variable clinical and immunological phenotypes. Unlike the European society for Immunodeficiency (ESID) criteria for diagnosis of MHC-II deficiency, most of them had lymphopenia with low T and B cells counts [27].

### CD40/CD40L deficiency

Three patients had CD40L deficiency, none had CD40 deficiency. Two sisters who had normal CD40 and elevated serum IgM, were then proved to have AICDA pathogenic variants when tested with NGS.

### Isolated CD4 lymphopenia

Among patients with isolated CD4 lymphopenia and variable serum immunoglobulin levels, 29 patients were found to have normal HLA-DR and DOCK8 expression whereas recent thymic emigrants (CD4+CD45RA+CD31+) were markedly reduced. Genetic testing done for some of these patients revealed two had RAG 1 mutations, three had DOCK2 mutations and two patients had CARD11 pathogenic variants.

## CID associated with syndromic features

### Wiskott aldrich syndrome (WAS)

Forty-seven patients were screened and 30 were diagnosed with WAS based on the results of FCM, thrombocytopenia and/or the low mean platelet volume. Some WAS patients showed decrease in CD3+T cells as part of the CID, however, this finding was not always present. Some mothers were tested, and none showed the bimodal pattern of the carrier.

### STAT3 deficiency

Three male patients were diagnosed to have STAT3 deficiency. Their diagnosis was confirmed by genetic analysis.

## Immunodeficiencies with predominantly antibodies defect

Twenty-two male patients were diagnosed with XLA. Their mothers showed the bimodal BTK expression on monocytes. Four of them were tested genetically and had hemizygous pathogenic variants in *BTK* gene. Twenty-seven patients were diagnosed to have CVID based on their manifestations, immunoglobin levels, and results of the FCM; of them, three had TACI deficiency, two had NFKβ2 deficiency and two had PIK3CD deficiency by genetic testing.

## Diseases of immune dysregulation

### LRBA deficiency

Ninety-nine patients with manifestations of immune dysregulation and immunodeficiency suggestive of LRBA deficiency were screened. Of those, 27 patients were diagnosed with LRBA deficiency; 17 (63%) were males and 10 (37%) were females. some of them were asymptomatic and screened being siblings of affected children.

### Autoimmune lymphoproliferative syndrome (ALPS)

Eleven patients (7 males and 3 females) were diagnosed to have ALPS based on the increase in CD3+TCRαβ+CD4−CD8−T cells. Diagnosis was confirmed for six patients by genetic testing.

### Perforin deficiency

Among 15 patients screened, two male patients were diagnosed with perforin deficiency.

### T regulatory cells defects

Tregs (CD4+CD25+FoxP3+) were assessed for patients with immune dysregulation. Thirty-three patients had defects in Tregs. However, for those patients, genetic analysis remains the only reliable method for definitive diagnosis.

## Defects of phagocytes

### Chronic granulomatous disease (CGD)

Between 2011 and 2021, 205 patients were diagnosed with CGD. The defective NADPH component was determined in 197 patients. The most common defective protein was p47*phox* (101 patients), followed by p22*phox* (49 patients), gp91*phox* (24 patients), p67*phox* (7 patients) and 16 male patients had gp91/p22*phox* deficiency, but their mothers were not tested*.*

### Leucocyte adhesion deficiency I (LADI)

Since 2011, when we started assessing CD18 and CD11, 35 patients (16 males and 19 females) were diagnosed with LAD I, all of them had < 2% of CD18− expressing neutrophils.

### *Congenital neutropenia*:

Thirty-four patients were diagnosed with congenital neutropenia (18 males and 16 females).

## Discussion

Diagnosis of IEI can be very challenging in settings of limited resources, high rate of infections and relatively high prevalence of PIDDs. In populations with high rate of consanguinity, IEI are not uncommon [7]. In 2015, CU PID center published its 5 years’ experience in screening and diagnosis of PIDDs [3], since then the number of PID patients followed up at the center has tripled as the referral to our center has increased The restricted accessibility to genetic testing, specially NGS, added to the challenge. To face this, we expanded the use of FCM in screening, diagnosis and follow up of PID patients. FCM was proved to be a powerful diagnostic tool in many IEI disorders while in others it was useful for screening and narrowing the possibilities of diagnoses [16, 18]. Protein expression assays increased the spectrum of the diagnoses achieved and helped reach definitive diagnosis in several disorders like, DOCK8 deficiency, LRBA deficiency, XLA, CGD, Perforin deficiency, ADA deficiency, IL7RA deficiency, LADI and MHC-II deficiency [12, 21, 22, 27]. Also, sending out for genetic testing has been cut down based on optimizing the molecular diagnosis strategy adapted in our center guided by the FCM [28]. Based on the FCM results, 744 patients (73%) were categorized and diagnosed with one of IEI disorders. In 271 patients, FCM was not useful and genetic testing was needed. These patients were categorized according to clinical manifestations, the results of genetic testing when available and ESID criteria. Most of those patients were diagnosed with defects of innate immunity including chronic mucocutaneous candidiasis, mendelian susceptibility of mycobacterial disease, disorders of bone marrow failure and complement defects.

For SCID patients, FCM screening allowed optimizing the use of genetic testing via targeted Sanger sequencing of certain genes. Following our algorithm in SCID screening, targeted sequencing of RAG1/2, ADA, IL7RA, JAK3, IL2RG, and PNP genes, allowed achieving the genetic diagnosis for families seeking prenatal diagnosis and significantly reduced the cost of genetic testing by restricting the need for NGS for certain cases. Targeted Sanger sequencing is more available and would cost less than third the cost of NGS per case in Egypt.

FCM analysis helped easy and definitive diagnosis of number of CID disorders. For example, *DOCK8* gene consists of 48 exons which makes the mutational analysis very difficult and troublesome [22]. When first started in 2014, and it took us 2 years to validate the FCM test results of DOCK8 deficiency expression assays due to limited accessibility to genetic testing [22]. Between 2014 and 2016, 15 patients were genetically diagnosed with DOCK8 deficiency and the FCM test was validated. In the next 5 years, 40 more DOCK8 patients were diagnosed based on the validated FCM test and other immunological features. Same applies on diagnosis of MHC-II deficiency and WAS syndrome. As for CD40L deficiency and STAT3 deficiency, FCM was a good screening test, narrowing the possibilities of the diagnosis. However, for those patients, genetic confirmation is indicated. For antibody defects, XLA was an example of how FCM provides successful precise diagnosis while for CVID, FCM was useful to screen the patients, fulfil probable diagnosis criteria and exclude LRBA patients.

Patients with immune dysregulation were tested for Tregs deficiency and LRBA expression. *LRBA* gene consists of 58 exons and can only be sequenced by NGS [12]. Performing the genetic testing for all the patients suspected to have LRBA deficiency in Egypt is not feasible. To validate the LRBA FCM expression assay test by means of genetic analysis, we genetically tested 18 LRBA patients along 2 years between 2017 and 2019 [12]. Since the test was validated, 99 patients with immune dysregulation were screened by FCM and 27 patients were proved to have LRBA deficiency. FCM allowed rapid and accurate diagnosis of those patients including asymptomatic patients’ siblings. Thirty-three patients had low Tregs percent/number, however their precise diagnosis requires genetic testing as many disorders with IPEX-like symptoms and multiple autoimmunity can be associated with decrease in Tregs [29].

FCM was effective in diagnosis of phagocytic defects. Two hundred-five patients were diagnosed with CGD. Protein expression assay of the NADPH components confirmed the diagnosis within hours from the initial testing with DHR. This also allowed identifying patients at higher risk of complications as patients with gp91*phox* and p22*phox* deficiencies tend to have more serious manifestations than patients with p47*phox* deficiency and need closer follow up [21, 30].

An example of how FCM helped us optimize genetic testing is an 8-years old male patient with absent B cells, normal T cells, thrombocytopenia and hypogammaglobulinemia. He was diagnosed as XLA for 5 years and was on IVIG. The patient was tested for BTK expression when the test was first introduced in our lab. He had normal expression of BTK which indicated genetic testing with NGS. The patient and his 3-years old sister, who had nonspecific symptoms and mildly decreased B cells, were diagnosed genetically with NHEJ deficiency. They were then transferred to transplantation unit for HSCT.

Assessment of T and B cell differentiation and protein expression assays were of benefit in follow up of patients who had HSCT. For example, DOCK8 patients who were transplanted usually regain normal protein expression and B cell differentiation within 3 months post-transplant. One of the earliest manifestations of graft versus host disease (GVHD) in one of the transplanted DOCK8 patients was regression in the DOCK8 protein expression level which became normal again with improvement of GVHD.

NGS sequencing of a group of patients with CID features associated with CD4 lymphopenia revealed DOCK2 deficiency in 3 patients and CARD11 deficiency in 3 patients. Hence, we plan to optimize FCM screening tests for intracellular staining of DOCK2 and CARD11 proteins. Some patients with manifestations suggestive of mendelian susceptibility of mycobacterial disease were genetically tested and 7 had pathogenic variants in IL12RB. Expression of IL12R by FCM can be useful for diagnosis of those patients specially that 22 patients with similar manifestations were not genetically tested yet.

Few centers have published their experience on using FCM as a diagnostic tool in IEI Reviews from India about FCM in IEI, showed that the commonest disorders diagnosed using FCM were HLH, SCID and CGD [17, 18]. In our center, the commonest disorders diagnosed using FCM were SCID, CGD, DOCK8 deficiency and other CID disorders. This difference is probably due to the difference in the inheritance pattern and ethnic origins. Our diagnosis spectrum was also different than other studies published from Egypt. Almalky and his colleagues reported that CID with syndromic features followed by antibody defects were the comments categories diagnosed among their cohort [31]. Another study from Egypt reported that antibody defects and combined immunodeficiencies were the highest categories diagnosed [32]. The differences are probably attributed to the larger cohort reported in the current study as well as the wider spectrum of the diagnostic tests used. Thus, optimizing the use of FCM in each center is crucial to reach utmost benefit of its use in IEI diagnosis.

## Conclusion

Multi-color FCM is a useful cost-effective tool for categorization, diagnosis and follow up of patients with IEI which are not rare in consanguineous populations. Optimization and validation of the tests are very important and should be done according to each center and types of IEI disorders encountered in each population. Although molecular testing remains the definitive diagnosis in some IEI disorders, however it is (Additional file 1: Table S1) not always available. Genetic testing can be tailored and advised in certain cases guided by the FCM results.

## Supplementary Information

**Additional file 1: Table S1:** A list of the reagents and monoclonal/polyclonal antibodies used in all the diagnostic tests performed at our lab.

## Data Availability

The data that support the findings of this study are available on request from the corresponding author, upon reasonable request.

## Abbreviations

IEI: Inborn errors of immunity
PID: Primary immunodeficiency
IUIS: International Union of Immunological Societies
MENA: Middle East and North Africa
FCM: Flow cytometry
ESID: European Society for Immunodeficiency
SCID: Severe combined immunodeficiency
IL-7R: Interleukin 7 receptor
JAK3: Janus Kinase 3
RAG: Recombination activation gene
ADA: Adenosine deaminase
PNP: Purine nucleoside phosphorylase
CID: Combined immunodeficiency
DOCK8: Dedicator of Cytokinesis 8
MHC-II: Major histocompatibility complex class II
AICDA: Activation Induced Cytidine Deaminase
DOCK2: Dedicator of Cytokinesis 2
CARD 11: Caspase recruitment domain-containing protein 11
WAS: Wiskott Aldrich syndrome
STAT3: Signal transducer and activator of transcription 3
XLA: X-linked agammaglobulinemia
BTK: Bruton tyrosine kinase
CVID: Common variable immunodeficiency
LRBA: Lipopolysaccharide (LPS)-responsive and beige-like anchor protein
CTLA-4: Cytotoxic T lymphocytes Antigen 4
ALPS: Autoimmune lymphoproliferative Syndrome
CGD: Chronic granulomatous disease
LAD: Leukocyte adhesion deficiency
