# Supplementary material for: Flow cytometry optimizing the diagnostic approach in inborn errors of immunity: experience from Egypt
Source: Allergy Asthma Clin Immunol. 2022 Jun 2;18:45. doi: 10.1186/s13223-022-00688-w (PMC9164555; doi:10.1186/s13223-022-00688-w)
Supplement: Supplementary file 1 — Additional file 1: Table S1: A list of the reagents and monoclonal/polyclonal antibodies used in all the diagnostic tests performed at our lab. [file 13223_2022_688_MOESM1_ESM.docx]

Additional file: Table S1:

| **Assay** | **Monoclonals and Reagents** |
| --- | --- |
| Follow up tube after hematopoietic stem cell transplantation | Anti-CD45-APC H7 (BD 560274)  Anti-CD3-APC (BD 345767)  Anti-CD56- PE (BD 345812)  Anti-CD19-PE-Cy7 (BD 341113)  Anti-CD8-FITC (BD 347313)  Anti-CD4-Percyp 5.5 (BD 552838)  Anti-CD45RA-BV510(BD 740186)  Anti-CD45RO-PE (BD 347967)  Anti-IgD-FITC (BD 555778)  Anti-CD27-BV421(BD 742731) |
| Basic Lymphocytes enumeration | Anti-CD3-ECD (UCHT1) (BC IM2705U)  Anti-CD4-FITC (13B8.2) (BC IM0448U)  Anti-CD8-PE (B9.11) (BC IM0452U)  Anti-CD19-PC5.1 (J3-119) (IM2643U)  Anti-CD56- PE-CY7 (N901) ( A51078) |
| IL-7RA expression | Anti-CD127-FITC (BD 560549) |
| ADA expression | Anti-CD3-PE-CY^TM^5 (BD555334)  Purified mouse monoclonal antibody to human ADA (Santa Cruz Sc-28346)  FITC-conjugated goat anti-mouse IgG/IgM antibody (BD 555988) |
| Memory and naïve T lymphocytes | Anti-CD4 –PC7 (Beckman Coulter 737660)  Anti-CD45RA –FITC (Beckman Coulter A07786)  Anti-CD45RO –PE (Beckman Coulter A07787) |
| Memory and naïve B lymphocytes | Anti-CD19 –PC5 (Beckman Coulter A07771)  Anti-CD27 –PC7 (Beckman Coulter A54823)  Polyclonal rabbit anti-Human IgD-FITC (Dako F0189) |
| Recent Thymic emigrants | Anti-CD4 –PC7 (Beckman Coulter 737660)  Anti-CD45RA –FITC (Beckman Coulter A07786)  Anti-CD31-PE (Beckman Coulter IM-2409) |
| Tregs | CD4-PE-Cy7 (Beckman coulter 737660)  Alexa Fluor® 647 Mouse anti-Human FoxP3 (BD561184)  CD25-PE (eBiosciences 12-0259) |
| DOCK8 expression | Purified rabbit anti-human DOCK8 (EPR12511) (Abcam ab175208)  F(ab)'2 Goat anti−Rabbit IgG-FITC (life technologies A24532) |
| MHC-II expression | Anti-CD19 –PC5 (Beckman Coulter A07771)  Anti human HLA-DR-PE (BD 560943) |
| CD40 expression | Anti-CD19 –PC5 (Beckman Coulter A07771)  Anti-CD40-PE (Beckman Coulter IM1936U) |
| CD40L expression | For stimulation:  Cell Stimulation Cocktail (plus protein transport inhibitors) (eBiosciences 00-4975)  For expression:  CD4-PE-Cy7 (Beckman coulter 737660)  Anti-CD154-FITC (BD 555699) |
| WASP expression | Purified mouse monoclonal antibody to human WASP (Santa Cruz Sc-13139)  FITC-conjugated goat anti-mouse IgG/IgM antibody (BD #555988) |
| P-STAT3 expression | For stimulation: Interleukin-6 (R&D 206-IL)  Alexa flour 488-conjugated pSTAT3 antibody (PS727, BD Bioscience cat.558085). |
| Th17 analysis | For stimulation:  Cell Stimulation Cocktail (plus protein transport inhibitors) (eBiosciences 00-4975)  Anti-Human IL-17F –PE Cy7 (eBiosciences 25-7169) |
| BTK protein expression | Anti-CD19 –PC5 (Beckman Coulter A07771)  Anti-CD14-PE (Beckman Coulter A07764)  Purified rabbit anti-human BTK (D3H5) (cell signaling 8547)  F(ab)'2 Goat anti−Rabbit IgG-FITC (life technologies A24532) |
| LRBA protein expression | Purified rabbit polyclonal antibody to LRBA (abcam ab121601)  F(ab)'2 Goat anti−Rabbit IgG-FITC (Life technologies A24532) |
| CTLA4 protein expression | For stimulation Ca+2 ionomycin (ab120116)  For CTLA4 assessment:  CD4-PE-Cy7 (Beckman coulter 737660)  CD45RO-PE (Beckman coulter A07787)  Alexa Fluor® 647 Mouse anti-Human FoxP3 (BD561184)  Purified anti-CD152 (BD Cat.550405) |
| Perforin expression | Anti-CD8-PE (Beckman Coulter A07757)  Anti-CD56-CY7 (BD 560916)  Anti-human Perforin-Alexa488 (BD 563764) |
| Double Negative T cells | Anti-CD3-PE (Beckman coulter no. A07747)  Anti-CD4-FITC (BD 340133)  Anti-CD8-FITC (BD 347313)  Anti-TCR-αβ PE/Cy5 (Abcam Ab95645). |
| DHR | Phorbol 12-myristate 13-acetate (PMA) (Sigma-Aldrich P8139)  Dihydrorhodamine- 123(Sigma-Aldrich 01054) |
| NADPH components expression | Purified rabbit monoclonal [EPR5065] to NOXA2/p67-phox (ab109523)  Purified rabbit monoclonal [ERP13134] to NCF1/p47-phox (ab179457),  Purified mouse monoclonal to cytochrome b245 light chain antibody/p22 (Sc-130551)  Purified rabbit polyclonal to NOX2/gp91-phox antibody (ab80508)  Alexa Fluor®647-conjugated goat anti-rabbit polyclonal antibody(ab181474)  FITC-conjugated goat anti-mouse IgG/IgM antibody (BD 555988) |
| LAD1 | CD18-PE (Beckman Coulter IM1570U)  CD11b-FITC (Beckman Coulter IM0530U). |
| Anti-neutrophil antibodies | Mouse anti-human IgG-FITC (BD 560952) |
| For permeabilization to assess intracellular protein expression | IntraPrep Permeabilization Reagent (Beckman Coulter IM2389) |
